# A RUBY-based *in vivo* hairy root induction system for rapid assessment of transformant and genome editing efficiency in potato

**DOI:** 10.1016/j.jgeb.2026.100673

**Published:** 2026-03-25

**Authors:** Linh Khanh Ly, Linh Khanh Chu, Cuong Xuan Nguyen, Oleg Stanislavovich Nikonov, Ekaterina Yu Nikonova, Ha Hoang Chu, Phat Tien Do

**Affiliations:** a Institute of Biology, Vietnam Academy of Science and Technology, Hanoi, Viet Nam; b Institute of Protein Research, Russian Academy of Sciences, Moscow, Russia; c Graduate University of Science and Technology, Vietnam Academy of Science and Technology, Hanoi, Viet Nam

**Keywords:** *Rhizobium rhizogenes*, CRISPR/Cas9, Hairy roots, RUBY, *Solanaceae*

## Abstract

Hairy root transformation systems are increasingly recognized as effective tools for functional genomics and gene editing studies in various crop species. In this study, we employed the *RUBY* reporter gene as a visible marker to establish a rapid, simple, and efficient *in vivo* system for potato hairy root induction and transformation. Explants were wounded by transverse cuts and gently scraped across the surface of a culture plate containing *Rhizobium rhizogenes* harboring the desired construct, then directly planted into pots containing moist, sterile vermiculite. The 2–3-week-old shoots regenerated from tubers of the Atlantic potato cultivar were identified as sufficient explants for *Rhizobium rhizogenes-*mediated transformation, achieving transformation efficiencies of up to 100%. This procedure was further adapted for stem nodes and leaf explants and achieved high transformation efficiencies of 80% and 93%, respectively. In addition, the *in vivo* hairy root transformation system was successfully utilized to validate CRISPR/Cas9 activities by inducing targeted mutations of the *StDL1* gene in potato. Heteroduplex analysis on PAGE revealed a high editing frequency (96%), and Sanger sequencing of selected lines confirmed diverse deletions at the target site. Overall, this *in vivo* hairy root transformation method enables rapid and robust assessment of genome editing efficiency and transgene expression, providing a valuable tool for functional genomics and genetic improvement in potato, with potential applications in other *Solanaceae* crops.

## 1 Introduction

Potato is an important food crop with high economic value and nutritional content. Following rice, wheat and maize, potato is the fourth most important food crop in the world in terms of production volume.^1^ This plant is considered an attractive crop for both subsistence and commercial farming due to its ease of vegetative propagation and high energy yield per unit area.^2^ However, potato cultivation faces increasing challenges caused by climate change. Biotic factors, including pests, nematodes, fungi, bacteria and viruses, along with abiotic factors such as high CO2 content, temperature, humidity and frost, negatively affect potato growth and yield performance.^3^, ^4^, ^5^, ^6^ These challenges demand innovative strategies to secure stable yields and enhance long-term agricultural resilience.

Conventional breeding methods have been effectively used to improve disease resistance and quality traits in potato. These approaches, however, are time-consuming and constrained by the crop’s complex tetraploid genome.^7^, ^8^ Genetic engineering has emerged as a powerful alternative, enabling the precise introduction, silencing, or editing of specific genes to generate potato lines with improved tolerance to both abiotic and biotic stresses.^9^, ^10^, ^11^, ^12^, ^13^ Despite its potential, gene editing in potato remains limited by low transformation efficiency, especially in commercial cultivars.^14^, ^15^. In plant genetic engineering and genome editing research, validating activities of transgene cassettes and genome editing systems is essential for downstream steps such as generating stable transgenic plants or creating mutant lines.^16^ In recent years, the *Rhizobium rhizogenes (R. rhizogenes)-*mediated hairy root transformation has been utilized as a powerful and rapid tool for assessing gene constructs and genome editing activities in various plant species.^17^, ^18^, ^19^ The integration of reporter genes such as *GUS* or *GFP* with CRISPR/Cas constructs in the *R. rhizogenes*-mediated transformation has significantly accelerated the selection of transgenic and mutant hairy roots.^19^, ^20^, ^21^, ^22^ This approach offers a time-saving and cost-effective platform for functional genomic and genome editing studies. Recently, the *RUBY* gene has been investigated and considered as a promising alternative visual reporter, with several advantages over traditional reporter genes like *GFP*, *GUS*, and luciferase.^23^ This color reporter gene is an artificial open-reading frame that encodes CYP76AD1, L-DOPA 4,5-dioxygenase and glucosyltransferase, which are key enzymes involved in the biosynthesis of betalain.^23^ Upon expression, the *RUBY* gene produces a red-colored pigment which can be used as a visual marker to select transformants without the need for biochemical or molecular analysis.^22^, ^24^, ^25^ RUBY-based expressed systems have been successfully demonstrated in *R. rhizogenes*-mediated transformation across various plant species.^26^, ^27^, ^28^

In this study, we established an efficient *R. rhizogenes*-mediated *in vivo* hairy root transformation system in potato. The *RUBY* was used as a visual reporter gene to assess the effects of potato cultivars and explant types on transformation efficiency. We further integrated RUBY with CRISPR/Cas9 constructs to facilitate the screening and validation of editing activity using the established hairy root transformation system. This study provides a promising platform to accelerate genetic engineering and genome editing research in potato, with potential applications across other *Solanaceae* crops.

## 2 Materials and methods

### 2.1 Plant materials and growth conditions

Three potato cultivars Atlantic, Marabel and Zhukovsky Early were maintained under *in vitro* conditions in MS20 medium (Table S1) at the Plant Cell Technology Laboratory, Institute of Biology, Vietnam Academy of Science and Technology. In addition, tubers of Atlantic were purchased from a local seedling company (VIETPO Corporation, Hanoi, Vietnam) and planted in plastic trays (60 cm × 35 cm × 15 cm) containing a substrate mixture of perlite and vermiculite at a 1:3 vol ratio. The generated potato plants were grown in an environment-controlled growth chamber (24 ± 2 °C, 16 h-light/8h-dark cycle, approximately 70% relative humidity) and fertilized with 1/10 MS solution (Table S1) to provide explants for bacterial inoculation.

### 2.2 Construction of CRISPR/Cas and RUBY expression system

**Fig. 1:**
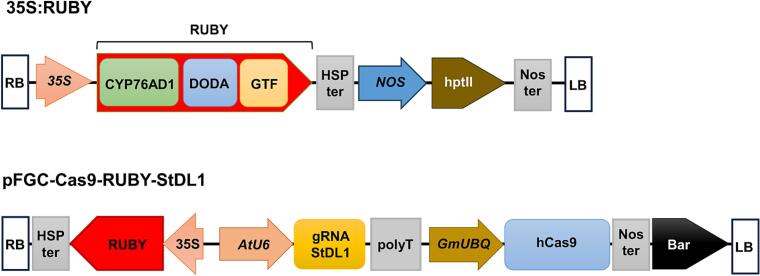
Constructs for potato hairy root transformation and genome editing. RB: right border; 35S: CaMV35S promoter; RUBY: a single open reading frame contains three genes CYP76AD1, DODA, and GTF, which are involved in the biosynthesis of betalain; HSP ter: heat shock protein terminator; Nos: nopaline synthase promoter; hptII: hygromycin resistance gene; Nos ter: nopaline synthase promoter terminator; AtU6: *Arabidopsis* U6 small nuclear RNA gene promoter; gRNA StDL1: guide ribonucleic acid for *StDL1* gene; polyT: poly-thymidine sequence; GmUBQ: *Glycine max* ubiquitin promoter; hCas9: Human codon-optimized Cas9 nuclease derived from *Streptococcus pyogenes*; Bar: phosphinothricin resistance gene; LB: left border.

To generate the pFGC-Cas9-RUBY-GW vector for cloning steps, the CaMV35S::RUBY::HSPter fragment was amplified from the 35S:RUBY construct (Addgene plasmid #160908) and inserted into the pFGC-Cas9-GW^29^ using NEBuilder® HiFi DNA Assembly Cloning Kit (E5520S, NEB, Ipswich, Massachusetts, USA). The gRNAs were designed using the CCTop online tool.^30^ A 23-nucleotide target sequence within the exon 1 region of the *StDL1* gene, which codes an R2R3 MYB transcription factor in potato, was used for CRISPR/Cas construction.^31^ The target sequence was synthesized by PhusaGenomics (Can Tho, Vietnam) as a pair of DNA oligonucleotides to be annealed to form dimers (Table S2). These dimers were cloned into the pAtU6-GW vector and subsequently transferred into the binary vector pFGC-Cas9-RUBY-GW at the attR1-attR2 sites using Gateway™ LR Clonase (Invitrogen, Waltham, Massachusetts, USA), resulting in the construct pFGC-Cas9-RUBY-StDL1 (Fig. 1). The complete construct was verified by Sanger sequencing and introduced into *R. rhizogenes* strain K599 via electroporation for potato hairy root transformation.

### 2.3 *R. rhizogenes*-mediated root transformation

**Fig. 2:**
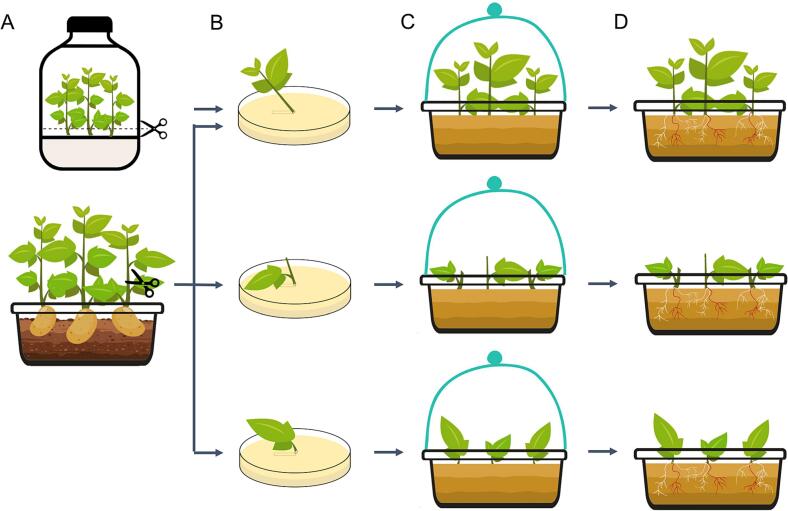
A procedure for potato hairy root transformation using *R. rhizobium-*mediated method. (A) The primary roots of the seedlings were cut with a sterile scissor. (B) At the wound site, gently scrape onto the surface of an *R. rhizogenes* culture harboring the target construct. (C) The infected explants were transferred directly to sterile, moist vermiculite-filled plastic trays and covered with transparent plastic domes to maintain high humidity. (D) The hairy root lines were generated 2 weeks after infection. Red roots represent transgenic roots and white roots represent non-transgenic roots. (For interpretation of the references to colour in this figure legend, the reader is referred to the web version of this article.)

The entire procedures for establishing potato hairy root transformation are illustrated in Fig. 2. Single bacterial colonies were incubated for 2 days at 28 °C in the dark on YEP solid medium supplemented with 50 mg/L kanamycin and 100 mg/L spectinomycin. Explants were collected from three-week-old *in vitro* plants or from 1 to 3-week-old plants generated from potato tubers and used for *R. rhizogenes* infection (Fig. 2A). Wounds were created by making oblique cuts on the internodes (for shoot and internode explants) or petioles (for leaf explants), followed by gentle scraping onto the surface of an *R. rhizogenes* culture harboring the target construct (Fig. 2B). The infected explants were then transferred to plastic trays containing moist, sterile vermiculite and covered with transparent plastic domes to maintain high humidity (Fig. 2C). No watering was applied during the first seven days post-inoculation. After this co-culture period, plants were monitored regularly and watered as needed to maintain appropriate moisture levels. The formation and frequency of hairy roots were assessed two weeks after infection (Fig. 2D). Subsequently, the explants were carefully removed from the substrate, and roots were washed gently with water. Transgenic hairy roots were identified by visible RUBY pigmentation. Numerous parameters were recorded at 2 weeks after infection, including the number of explants with hairy roots, the number of hairy roots per wound site, the number of explants with RUBY hairy roots, the number of RUBY hairy roots per explant, and the average transgenic hairy root length.

### 2.4 Validation of CRISPR/Cas9 activities

Independent transgenic root lines were collected based on RUBY expression, and total DNA was extracted using the CTAB method.^32^ The expanded target region of the *StDL1* gene was amplified using specific primers (StDL1-F/R). PCR was carried out with DreamTaq Green PCR Master Mix (Cat. No. K1081 – Thermo Scientific, Waltham, Massachusetts, USA), with the following thermal cycling condition: an initial denaturation at 95 °C for 3 min; followed by 35 cycles of 95 °C for 20 s, 58 °C for 20 s, and 72 °C for 30 s; and a final extension at 72 °C for 5 min. To identify CRISPR/Cas9-induced mutations in the target gene, PCR products from each transgenic root line were mixed with PCR products from the wild-type sample (Atlantic potato leaves) at a 1:1 ratio. The mixtures were denatured at 95 °C for 5 min and then cooled to room temperature to form the heteroduplex and homoduplex DNA fragments. The resulting PCR products were analyzed by the PAGE method.^19^, ^33^, ^34^ Mutations were detected based on DNA shifted bands between the mutant and WT lines on the PAGE. PCR amplicons from the mutant hairy root lines were purified and ligated into the cloning vector pJET1.2 (Thermo Fisher Scientific, Waltham, Massachusetts, USA) for Sanger sequencing. Sequencing was performed using the Applied Biosystems Big Dye Terminator cycle sequencing kit (Applied Biosystems, Waltham, Massachusetts, USA) on ABI PRISM R 3100 Avant Genetic Analyzer system. The sequences were analyzed using the MUSCLE 3.8.31 program.

### 2.5 Statistical analysis

The hairy root induction rate was calculated as the number of explants that formed hairy roots per infected explant. Transgenic roots were identified by the presence of red coloration (RUBY expression). Transformation efficiency was determined as the percentage of explants with RUBY roots to the total number of infected explants. Each experiment was performed with at least three replicates. Data were analyzed using Duncan’s multiple range test (DMRT) in SPSS software version 20. Results are presented as mean values ± standard deviation (SD).

## 3 Results

### 3.1 Establishment of an *in vivo* hairy root transformation system for potato with the *RUBY* gene

#### 3.1.1 Preliminary screening of potato genotypes for hairy root induction and transformation

**Fig. 3:**
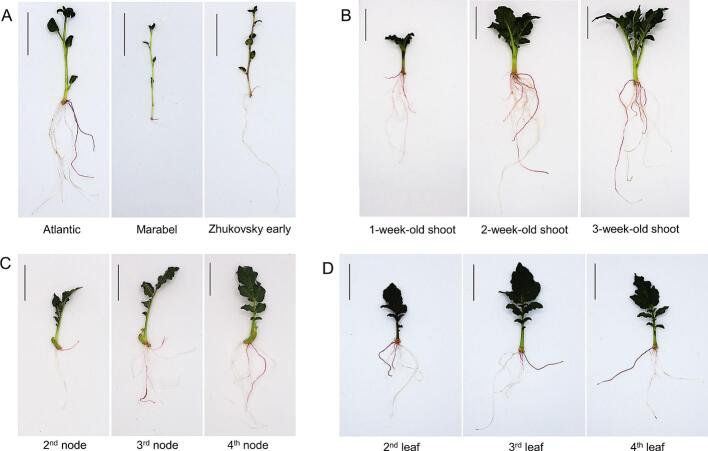
Effect of plant materials on the hairy root transformation efficiency. (A) Different potato cultivars. (B) Different plant ages. (C) Different potato stem node segments. (D) Different potato leaves. Scale bar = 3 cm.

To identify potential genotypes for potato hairy root, *in vitro* shoots from three cultivars (Atlantic, Marabel, and Zhukovsky Early) were used as explants for *R. rhizogenes* infection. Hairy roots were successfully induced from the explants of all genotypes, as infected with *R. rhizogenes* strain K599 harboring the 35S:RUBY construct (Table 1). The hairy root induction rates were observed at 100% for all tested potato varieties. Transgenic hairy roots were visually observed by red pigmentation conferred by RUBY expression. The three potato genotypes exhibited significant differences in the total number of induced hairy roots, hairy root length, and transformation efficiency. In particular, the transformation efficiencies of the Atlantic and Zhukovsky cultivars reached 44.4% and 40.0%, respectively, whereas the efficiency in the Marabel cultivar was markedly lower at 22.2%, representing a twofold reduction. Atlantic also displayed the highest number of hairy roots (7.09 roots/explant) compared to Marabel and Zhukovsky Early (4.62 and 4.53 roots/explant, respectively). Notably, root length varied substantially among genotypes (Fig. 3A). Atlantic showed the longest RUBY-expressing hairy roots (4.25 cm), whereas the shortest roots (0.53 cm) were observed in the Marabel. These differences may be associated with the thin-stem morphology of the Marabel cultivar, which was observed in both *in vitro* and greenhouse conditions. Overall, the Atlantic cultivar exhibited the highest transformation efficiency across all evaluated parameters, including transformation efficiency, number of hairy roots induced per wound site, the number of RUBY hairy roots, and the average transgenic hairy root length. Therefore, Atlantic was selected as a sufficient material for subsequent experiments in this study.

**Table 1:** Hairy root transformation efficacies of different potato cultivars.

Cultivars	Total induced roots (per plant)	Total RUBY transgenic roots (per plant)	The RUBY transgenic root length (cm)	The hairy root induction rate (%)	The RUBY transformation efficiency (%)
Atlantic	7.09 ± 1.41^a^	1.79 ± 0.42^a^	4.23 ± 1.24^a^	100.00 ± 0.00^a^	44.44 ± 7.70^a^
Marabel	4.62 ± 1.00^b^	1.25 ± 0.25^a^	0.53 ± 0.32^b^	100.00 ± 0.00^a^	22.22 ± 7.70^b^
Zhukovsky early	4.53 ± 0.50^b^	1.19 ± 0.20^a^	1.28 ± 0.53^b^	100.00 ± 0.00^a^	40.00 ± 6.67^a^

Values represent the mean ± SD for three independent replicates (n = 15), whereas different letters (a and b) indicate significant differences (p < 0.05) using ANOVA and DMRT tests.

#### 3.1.2 Assessments of potato seedling ages on hairy root induction

To evaluate the impact of plant ages on hairy root induction and transformation efficiency, 3-cm shoot segments were cut from potato plants generated from sprouting tubers at one, two, and three weeks. These shoot segments were used as explants for *R. rhizogenes* inoculation. 2 weeks post-infection, all shoot segments (100%) from 1-, 2- and 3-week-old plants formed hairy roots (Table 2, Fig. 3B). The total hairy roots per sample increased with plant ages, from 10.13 in 1-week-old plants to 17.87 in 3-week-old plants. A similar trend was also observed with the number of RUBY hairy roots per explant. Importantly, the transformation efficiency (100%) was highest in the 2- and 3-week-old plants, but decreased to 93.33% in the 1-week-old plants. Moreover, the composite plants (plants with hairy roots) produced from the 2- and 3-week-old explants grew faster with healthy leaf and root systems (Fig. 3B). Considering both transformation performance and time efficiency, the 2-week-old plants were identified as the optimal materials for potato hairy root transformation.

**Table 2:** Effects of plant ages on the hairy root transformation efficacy.

Plant ages	Total induced roots (per plant)	Total RUBY transgenic roots (per plant)	The hairy root induction rate (%)	The RUBY transformation efficiency (%)
1-week-old shoots	10.13 ± 1.81^b^	2.83 ± 0.50^b^	100.00 ± 0.00^a^	93.33 ± 5.77^a^
2-week-old shoots	16.90 ± 3.14^a^	5.93 ± 0.76^a^	100.00 ± 0.00^a^	100.00 ± 0.00^a^
3-week-old shoots	17.87 ± 2.82^a^	5.73 ± 1.36^a^	100.00 ± 0.00^a^	100.00 ± 0.00^a^

Values represent the mean ± SD for three independent replicates (n = 10), whereas different letters (a and b) indicate significant differences (p < 0.05) using ANOVA and DMRT tests.

#### 3.1.3 Extending materials for potato hairy root transformation

To extend material resources for potato hairy root transformation, stem node and leaf explants of the 2-week-old potato plants were used for *R. rhizogenes* infection. The hairy root formed from the wounding sites of all tested explants (leaves and nodes) two weeks post-inoculation (Fig. 3C, D). For the node explants, no significant difference in the transformation efficiency was observed across node positions, as all showed 70% or higher rates (Table 3). Although lower nodes tended to produce slightly more roots and RUBY roots per explant, the differences were not pronounced. Interestingly, lateral shoots emerged from the node explants at two weeks post-inoculation and could develop into composite potato plants (Fig. 3C). For leaf explants, the younger leaves (leaves near the apex) showed higher capacity for hairy root transformation. Both the total induced roots and the transformation efficiency tended to decline with leaf ages, while the total induced hairy roots per explant showed significant differences. Specifically, the 2nd leaves produced more hairy roots (11.27) than the 3rd and 4th leaf explants (7.10 and 6.13, respectively) (Table 4 and Fig. 3D). The number of RUBY hairy roots per explant showed less variation with around 2 red roots per leaf. Thus, although less effective than the shoot segments, node and leaf explants could be used as materials for potato hairy root transformation.

**Table 3:** Hairy root transformation efficacies of different stem node segments.

Node explants	Total induced roots (per plant)	Total RUBY transgenic roots (per plant)	The hairy root induction rate (%)	The RUBY transformation efficiency (%)
2nd nodes	6.50 ± 1.04^a^	1.3 ± 0.45^a^	100.00 ± 0.00^a^	73.33 ± 5.77^a^
3rd nodes	9.17 ± 3.29^a^	1.63 ± 0.68^a^	100.00 ± 0.00^a^	70.00 ± 10.00^a^
4th nodes	9.97 ± 3.17^a^	2.7 ± 1.65^a^	100.00 ± 0.00^a^	80.00 ± 17.32^a^

Values represent the mean ± SD for three independent replicates (n = 10), whereas the same letter (a) indicates no significant differences (p < 0.05) using ANOVA and DMRT tests.

**Table 4:** Hairy root transformation efficacies of different potato leaves.

Leaf explants	Total induced roots (per leaf)	Total RUBY transgenic roots (per leaf)	The hairy root induction rate (%)	The RUBY transformation efficiency (%)
2nd leaf	11.27 ± 2.99^a^	2.66 ± 0.70^a^	100.00 ± 0.00^a^	93.33 ± 5.77^a^
3rd leaf	7.10 ± 1.32^b^	2.06 ± 0.65^a^	100.00 ± 0.00^a^	76.67 ± 11.55^a^
4th leaf	6.13 ± 0.71^b^	2.19 ± 0.55^a^	100.00 ± 0.00^a^	70.00 ± 17.32^a^

Values represent the mean ± SD for three independent replicates (n = 10), whereas different letters (a and b) indicate significant differences (p < 0.05) using ANOVA and DMRT tests.

### 3.2 Application of *R. rhizogenes* mediated transformation to validate CRISPR/Cas construct activities in potato

**Fig. 4:**
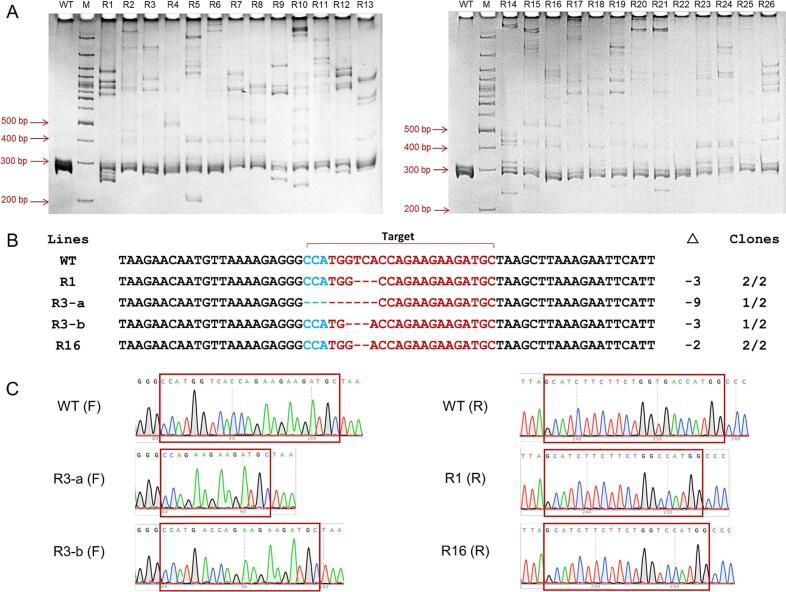
Validation of CRISPR/Cas9 activities via potato hairy root transformation. (A) Identification of *StDL1-*targeted mutations of transgenic hairy roots using heteroduplex analysis on PAGE. WT: Wild-type hairy roots, R1-R26: Transgenic hairy roots, M: GeneRuler 100 bp Plus DNA Ladder (Thermo Scientific™, Waltham, Massachusetts, USA). (B) Sanger sequencing of expanding target sites in the *StDL1* of transgenic hairy root lines. Red letters indicate target sites, blue letters indicate PAM sequences. Δ indicates targeted sequence changes in the allele, – for deletion. (C) Sequencing chromatograms of WT and mutant hairy root lines. The target region is marked by a red rectangle. F: sense strand sequence, R: antisense strand sequence. (For interpretation of the references to colour in this figure legend, the reader is referred to the web version of this article.)

In this study, we integrated RUBY expression cassette with the CRISPR/Cas9 system to evaluate the efficiency of potato hairy root transformation. Twenty-six independent transgenic hairy root lines expressing the *RUBY* reporter gene were randomly selected to assess the CRISPR/Cas9-induced mutations of the targeted gene (*StDL1*). The PAGE analysis revealed that 25 out of 26 root lines (96%) exhibited DNA shifted bands compared to the WT control, indicating mutations presented in the targeted sites of the *StDL1* gene (Fig. 4A). Subsequently, three hairy root lines were randomly selected for further Sanger sequencing analysis. The sequencing results were consistent with the PAGE analysis, exhibiting deletions at the target site of the *StDL1* gene. Importantly, all three lines exhibited distinct indels ranging in size from −2 bp to −9 bp (Fig. 4B, C). These results demonstrate that the established *in vivo* hairy root transformation system is highly effective for validating CRISPR/Cas construct activities in potato and has potential application in other *Solanaceae* plants.

## 4 Discussion

The *R. rhizogenes-*mediated transformation has been developed and widely applied as one of the most rapid and efficient methods for validating gene expression constructs and genome editing systems across various plant species. This method enables the rapid generation of transgenic roots, typically within approximately two weeks post-inoculation.^35^, ^36^ Each hairy root line represents an independent and stable transformant event, and in many plant species, these roots are capable of regenerating into whole transgenic plants.^19^, ^37^, ^38^ The *R. rhizogenes*-mediated transformation has been widely conducted under *in vitro* conditions, which are often labor-intensive and technically demanding due to the strict requirements for aseptic conditions. Recently, the successful implementation of hairy root systems under non-sterile conditions in various plant species offers a promising alternative to overcome these limitations.^17^, ^39^ The *in vivo* systems provide significant advantages, including minimal equipment needs and reduced reliance on clean rooms or highly specialized technical skills. However, the *R. rhizogenes-*mediated hairy root method has not been developed for potato under *in vivo* conditions. In this study, we established a one-step *R. rhizogenes-*mediated hairy root transformation that allows for the generation of composite potato plants within approximately two weeks post-inoculation. Successfully transformed hairy roots carrying the *RUBY* gene can be visibly distinguished by their red pigmentation, allowing rapid screening without molecular analysis. This protocol is compatible with non-sterile explants and can achieve transformation efficiencies of up to 100% with optimal materials. This rate is comparable to the highest *in vitro* transformation rates previously reported for *R. rhizogenes*-mediated transformation on tuber discs of the model potato cultivar Désirée,^40^ and substantially exceeds the transformation efficiency observed with *R. tumefaciens*-mediated methods under *in vitro* conditions in the Atlantic cultivar.^14^ This procedure is potentially for rapid gene function analysis, promoter activity evaluation, or high-throughput screening of gene editing systems in potato.

In other *R. rhizogenes*-mediated hairy root transformation systems, shoot tips or seedlings were commonly used as explants for bacterial infection due to their high regenerative capacity.^39^, ^41^, ^42^ However, in the plant species with limited seed production or reduced branches like potato, the availability of these types of explants can be a challenge. Recent studies have successfully utilized alternative plant tissues such as leaves and stem segments as materials for *R. rhizogenes*-mediated hairy root transformation. However, the transformation efficiency varied highly depending on the explant types and plant species.^17^, ^22^, ^42^ For instance, transgenic hairy roots were successfully induced from stem segments of *Plukenetia volubilis* at a low rate of 3.49% after 30 days post-inoculation,^22^ whereas leaf explants of *Idesia polycarpa* achieved higher hairy root induction and transformation rates, at 68.25% and 53.48%, respectively.^42^ In this research, we examined different materials including shoot tips, node segments, and leaf explants of potato seedlings generated from tubers for *R. rhizogenes*-mediated transformation under *in vivo* conditions. The high hairy root induction rates (100%) were achieved with all three types of explants, indicating the potential of these materials for *R. rhizogenes*-mediated transformation in potato. In addition, the high transformation efficiencies (≥70%) obtained with leaf explants and node segments of potato were higher than other crops. These results indicate that transgenic hairy roots can be efficiently generated from various materials of potato plants, offering a promising tool for further genetic engineering studies.

The *in vivo* hairy root transformation system and the generation of composite plants have been widely utilized in studies of gene function,^17^, ^22^ plant-environment interactions^43^, ^44^ and promoter functional analysis.^39^, ^45^ More recently, this system has been effectively and broadly applied for evaluating the activity of genome editing constructs.^19^, ^22^, ^38^ In this study, we successfully employed an *in vivo* hairy root induction system to evaluate the mutagenic efficiency of CRISPR/Cas9-mediated editing of an endogenous gene in potato. The high editing efficiency and the diversity of mutations in hairy roots demonstrate the effectiveness of the *in vivo* hairy root transformation system in screening and selecting gene-editing constructs in potato. Despite of the sequenced genome,^46^, ^47^, ^48^ the functions of many genes in potato remain unknown.^49^ Therefore, the *in vivo* hairy root transformation system developed in this study emerges as a powerful tool to accelerate functional genomics research in potato in the future.

## 5 Conclusion

In this study, the *RUBY* gene was utilized to establish a simple, rapid and efficient system for *R. rhizogenes*-mediated hairy root transformation in potato under non-sterile conditions. The high hairy root transformation efficiencies were achieved with various potato plant materials, indicating their potential for further applications. In addition, this non-sterile hairy root transformation system was successfully applied to validate the activity of a CRISPR/Cas9 system for targeting mutations of endogenous genes in potato. This result provides a foundation for further genetic engineering and genome editing studies in potato, with potential application across other *Solanaceous* species.

## CRediT authorship contribution statement

**Linh Khanh Ly:** Writing – original draft, Investigation, Formal analysis, Data curation. **Linh Khanh Chu:** Visualization, Investigation. **Cuong Xuan Nguyen:** Data curation, Conceptualization. **Oleg Stanislavovich Nikonov:** Writing – review & editing. **Ekaterina Yu Nikonova:** Writing – review & editing. **Ha Hoang Chu:** Writing – review & editing. **Phat Tien Do:** Writing – review & editing, Supervision, Project administration, Methodology, Conceptualization.

## Declaration of competing interest

The authors declare that they have no known competing financial interests or personal relationships that could have appeared to influence the work reported in this paper.
