# Supplementary material for: A RUBY-based in vivo hairy root induction system for rapid assessment of transformant and genome editing efficiency in potato
Source: J Genet Eng Biotechnol. 2026 Mar 25;24(2):100673. doi: 10.1016/j.jgeb.2026.100673 (PMC13053746; doi:10.1016/j.jgeb.2026.100673)
Supplement: Supplementary Data 1 [file mmc1.docx]

**Appendix A. Supplementary data**

**Table S1:** Media used in this study

| **Medium** | **Composition or references** |
| --- | --- |
| MS | Murashige and Skoog, 1962 |
| MS20 | MS, 20g/L sucrose and 7.5g/L agar; pH 5.8 |
| 1/10MS | 1/10MS without vitamins |
| YEP | 10 g/L yeast extract + 10 g/L bacto peptone + 5 g/L NaCl + 15 g/Lbacto agar; pH 7.0 |

**Table S2:** List of primers used in this study

| **Primers** | **Sequence of primers (5’ 🡪 3’)** | **PCR product size** |
| --- | --- | --- |
| F-StDL1-crispr | GATTGCATCTTCTTCTGGTGACCA |  |
| R-StDL1-crispr | AAACTGGTCACCAGAAGAAGATGC |  |
| F-stDL1geno | ATGGGAAGAGCTCCTTGTTGTG | 246 bps |
| R-stDL1geno | GTCTACGTATAACATCCTATCC |  |
| F-pJET | CGACTCACTATAGGGAGAGCGGC |  |

**Supplemental references**

Murashige T, Skoog F (1962) A revised medium for rapid growth and bio assays with tobacco tissue cultures. Physiologia plantarum 15: 473–497. doi:10.1111/j.1399-3054.1962.tb08052.x
